# Strangulation du pénis: à propos de deux cas en milieu rural

**DOI:** 10.11604/pamj.2018.30.128.14362

**Published:** 2018-06-13

**Authors:** Ibrahima Dara Damé, Ibrahima Diallo, Modou Ndiaye, Mamadou Beye, diaga Seck Ndour, Tiennou Hafing, Samba Thiapato Faye, Ibrahima Bocar Welle, Yaya Sow, Boubacar Fall

**Affiliations:** 1 Centre Hospitalier Regional d’Ourossogui, Ourossogui, Senegal; 2 Centre Hospitalier Regional de Ziguinchor, Ziguinchor, Senegal; 3 Centre Hospitalier Abass Ndao, Dakar, Senegal; 4 Centre Hospitalier Universitaire Aristide Le Dantec, Dakar, Senegal

**Keywords:** Strangulation, schizophrènes, urgences, pénis, Strangulation, schizophrenics, emergencies, penis

## Abstract

La strangulation du pénis est une pathologie nécessitant une prise en charge en urgence. Plusieurs objets métalliques ou non, peuvent être placés sur le pénis pour augmenter les performances sexuelles ou pour des fins auto-érotiques. Nous rapportons les observations de deux patients schizophrènes de 25 et 33ans reçus aux urgences pour une strangulation du pénis par anneau métallique. L'anneau était placé au niveau du sillon balanopénien depuis 3 jours chez l'un et au niveau de la racine de la verge depuis 2 jours chez l'autre. Il n'y avait pas de trouble urinaire chez les deux patients. L'ablation de cet anneau a été réalisée par taxis sous anesthésie locale chez l'un et sous sédation à la fentanyl suivie d'une double section de l'anneau à l'aide d'un engin électrique dans le 2^ème^ cas. A travers cette observation nous rapportons la prise en charge non chirurgicale de ces cas pathologique.

## Abstract (en)

Penile strangulation is a clinical condition requiring emergency management. Several metallic or non-metallic objects can be placed around the penis to improve sexual performance or for auto-erotic purposes. We report the case of two schizophrenic patients aged 25 and 33years hospitalized in the Emergency Department due to penile strangulation by metal ring. The ring was placed at the level of the glans foreskin groove 3 days before in one case and at the level of the root of penis 2 days before in the other. There was no urinary disorder in both patients. Ring ablation was performed with taxis under local anesthesia in one case and under sedation followed by double ring section using an electric saw in the other case.

## Introduction

La strangulation du pénis a été rapportée pour la première fois par Gauthier M. en 1755 [1]. Jusqu'à maintenant de nombreux articles ont été publiés avec divers dispositifs strangulant. Le tableau est souvent rencontré chez des patients psychologiquement déséquilibrés se livrant à des pratiques d'automutilation. Chez d'autres le motif est soit l'auto-érotisme soit le désire d'améliorer la performance sexuelle par une rigidité durable [2].

## Patient et observation

**Figure 1:**
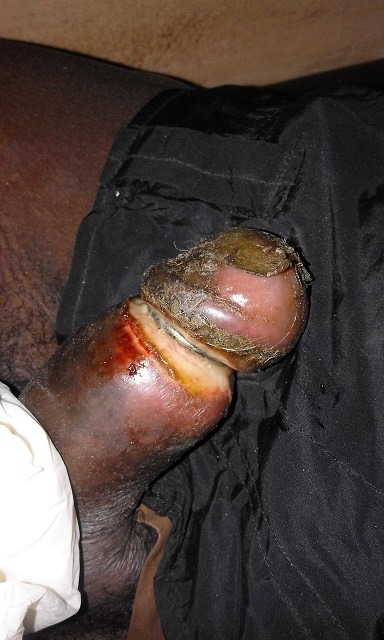
Strangulation de la verge par une bague métallique enfouie au niveau du sillon balano-prépucial

**Figure 2:**
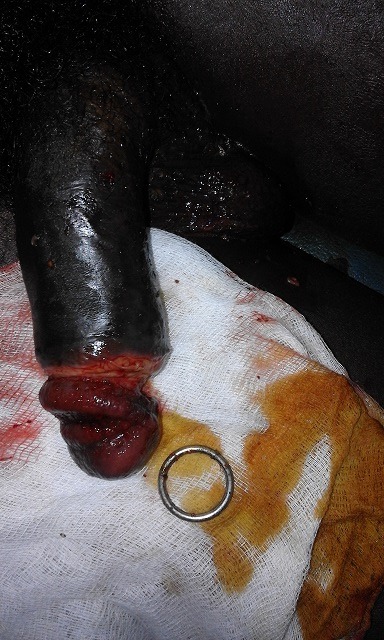
Recoloration du gland après l’ablation de l’anneau métallique

**Observation 1:** Monsieur H.D âgé de 25ans, célibataire, schizophrène sous neuroleptiques (chlorpromazine, halopéridol et trihexyphenidyle) est admis aux urgences pour une strangulation de la verge par un anneau métallique depuis 3jours. L'examen physique retrouvait un anneau métallique au niveau du sillon balanoprépucial étranglant la verge. On notait par ailleurs un important œdème du gland avec une plaie suppurée (Figure 1). La partie distale de la verge était souple. Le gland était engorgé pale, avec une perte totale de la sensibilité. On note par ailleurs l'absence de fistule urétrocutanée et de rétention d'urine. L'ablation de cet anneau a été faite sous anesthésie locale. Le geste a consisté en une réduction par taxis dont le principe consistait en une compression douce et prolongée sur le gland tout en poussant en avant l'anneau. Le patient avait bénéficié de soins locaux au niveau de la plaie et a été adressé par la suite en psychiatrie pour prise en charge. A l'ablation de cet anneau le gland s'est recoloré et on notait une plaie à la face ventrale du sillon balanopénien mais sans atteinte urétrale (Figure 2). Des soins locaux avec un décapage des lésions cutanées ont été réalisés, les suites étaient marquées par un épisode isolé de rétention aigue d'urine à J1 ayant nécessité un drainage vésicale transurétral. Le patient a évolué bien sous antibiothérapie et soins Locaux et est adresse en psychiatrie pour prise en charge.

**Figure 3:**
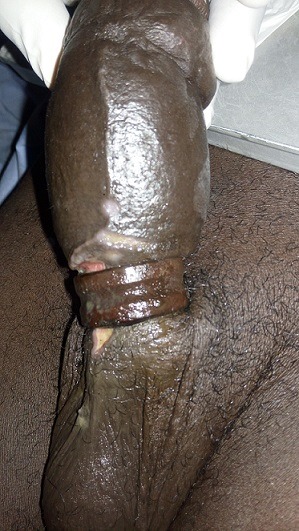
Strangulation de la verge par un anneau métallique

**Observation 2:** Monsieur YD âgé de 33 ans célibataire, schizophrène suivi est admis pour une strangulation de la verge par un anneau métallique évoluant depuis 2jours. L'examen mettait en évidence un anneau métallique en acier à la base de la verge et un important lymphœdème de la verge. Il n'y avait pas de signes d'ischémie ni de nécrose pénienne en aval de l'anneau. L'ablation de l'anneau a été immédiatement faite sous anesthésie au masque par double section de l'anneau à l'aide d'un engin électrique (Figure 3). A l'ablation de l'anneau les corps caverneux étaient à nu sans lésion spongieuse. Le patient a bénéficié de soins locaux de la plaie de la racine de la verge et d'un transfert en psychiatrie pour suite de prise en charge. Les suites ont été simples.

## Discussion

La strangulation pénienne par un anneau métallique est un traumatisme grave rencontré en particulier, chez des patients psychologiquement déséquilibrés [3] comme retrouvé chez nos deux patients. Le motif de ce geste volontaire est soit l'auto-mutilation [3,4] soit L'auto-érotisme ou même le désir d'améliorer la performance sexuelle par une rigidité pénienne plus durable [5]. Nombreux sont les dispositifs bloqués à la base de la verge. II peut s'agir d'anneaux métalliques (bague, anneau de rideau, porte-clés …) ou non métalliques (collet de bouteille plastique …) [4]. Dans tous les cas il requiert une décompression en urgence. Le retour veineux et lymphatique est interrompu en premier, engendrant l'œdème qui apparait au bout de quelques heures et gène l'extraction de l'anneau. Si la compression évolue, le flux artériel peut être compromis [4,6]. Le traitement doit être urgent [7]. La première étape consiste en l'ablation du matériel compressif souvent sous anesthésie locale. La technique utilisée dépend de la dureté et de la forme de lanneau. Pour les anneaux peu épais et peu larges, la section est généralement facile, faite à l'aide de pinces coupantes. En revanche ce geste est difficile pour les anneaux durs épais et/ou larges comme chez notre 2^ème^ patient qui a nécessité une sédation et l'usage d'un engin électrique pour sectionner l'anneau [4]. Il est recommandé dans ces cas de drainer le sang stagnant dans la verge par incision ou ponction du gland suivie d'un bobinage compressif de la verge par un fil de soie sur lequel est glisse progressivement l'anneau strangulant [7-9]. Ces gestes se sont avérés inefficaces chez notre 2^ème^ patient du fait de la sévérité de la strangulation et de l'importance du lymphœdème. Chez notre 1^er^ patient l'ablation de l'anneau métallique a été faite sous anesthésie locale à l'aide d'un geste qui a consisté en une réduction par taxis comme on le ferait devant un paraphimosis (compression douce et prolongée sur le gland tout en poussant en avant l'anneau). En effet la localisation distale de l'anneau au niveau du sillon balono-glanique a facilité cette manœuvre.

## Conclusion

La strangulation de la verge par anneau métallique ou non est facilement diagnostiquée. Les patients psychologiquement et sexuellement atteints constituent le terrain de prédilection. L'ablation urgente de l'anneau est la première étape thérapeutique. Cette prise en charge dépend surtout du raisonnement et du bon sens de l'urologue et des moyens dont il dispose.

## Conflits d’intérêts

Les auteurs ne déclarent aucun conflit d'intérêts.
